# A Crowdsourcing Open Contest to Design Pre-Exposure Prophylaxis Promotion Messages: Protocol for an Exploratory Mixed Methods Study

**DOI:** 10.2196/15590

**Published:** 2020-01-03

**Authors:** Jordan J White, Allison Mathews, Marcus P Henry, Meghan B Moran, Kathleen R Page, Carl A Latkin, Joseph D Tucker, Cui Yang

**Affiliations:** 1 Center for Public Health and Human Rights Department of Epidemiology Johns Hopkins Bloomberg School of Public Health Baltimore, MD United States; 2 Community Expert Solutions, Inc Durham, NC United States; 3 Know Your Place Movement JOY Baltimore Baltimore, MD United States; 4 Department of Health, Behavior, and Society Johns Hopkins Bloomberg School of Public Health Baltimore, MD United States; 5 School of Medicine University of North Carolina at Chapel Hill Chapel Hill, NC United States

**Keywords:** crowdsourcing, HIV, pre-exposure prophylaxis (PrEP), sexual and gender minorities

## Abstract

**Background:**

In the United States, black men who have sex with men (BMSM) are disproportionately affected by HIV. Pre-exposure prophylaxis (PrEP) can reduce HIV incidence. However, real-world implementation of PrEP outside of clinical trials has identified racial disparities in PrEP awareness, uptake, and adherence. In the context of a long history of medical mistrust and power imbalances between scientists and community members, strategies to increase uptake of PrEP among BMSM should consider ways to ensure messages address the needs and priorities of the community. Crowdsourcing contests shift traditional individual tasks to a large group and may enhance community engagement.

**Objective:**

This paper describes the research protocol of a contest approach to soliciting PrEP promotion messages among BMSM in Baltimore.

**Methods:**

Open-contest implementation and evaluation will proceed as follows: (1) organize a community steering group; (2) develop platforms to solicit crowd input; (3) engage the community to contribute ideas through a Web-based forum and in-person events; (4) evaluate contest entries using both community panel judge assessment and crowd voting; (5) utilize mixed methods to evaluate feasibility, acceptability, and community engagement; and (6) disseminate contest results.

**Results:**

This study was funded by the National Institutes of Health (National Institute of Mental Health: R34MH116725) in May 2018 and was approved by the institutional review board in April 2018. The open contest started in February 2019, and data analyses for the mixed method evaluation are expected to complete in December 2019.

**Conclusions:**

The contest will potentially bring new ideas in developing more impactful and locally defined PrEP promotion campaigns. We will determine whether an open-contest approach is acceptable among BMSM in Baltimore. If successful, this study can inform future projects using a similar approach on how to identify and implement programs and policies that are more responsive to community needs and that build up community assets.

**International Registered Report Identifier (IRRID):**

DERR1-10.2196/15590

## Introduction

Despite the development of efficacious biomedical HIV prevention and treatment methods, the history of HIV/AIDS includes many examples in which most vulnerable populations have not equally benefited from proven biomedical interventions [1,2]. Significant racial and ethnic disparities in new HIV infections persist in the United States [3]. Black men who have sex with men (BMSM) have the highest rate of HIV infection in the United States, accounting for over half of all new HIV infections each year [4]. The US Federal Initiative to End the HIV Epidemic recommends enhancing pre-exposure prophylaxis (PrEP) uptake among those most at risk for HIV transmission in the United States [5]. However, emerging studies of populations at risk of HIV in the United States have identified racial disparities in PrEP awareness, uptake, and adherence [6-8]. Major social and structural barriers for PrEP uptake, such as stigma and mistrust of the health system [9], are often unaddressed, especially among racial minority men who have sex with men (MSM) [10]. Power dynamics between researchers and community members infrequently provide meaningful community engagement, ownership, and authorship in the process of research and program development, which create barriers to acquiring information that could drive better decision making for HIV program development and implementation. Research indicates stigma and mistrust of the health system as possible barriers to taking PrEP [9], especially among minority MSM [10]. This is problematic because minority MSM would benefit most from HIV-preventative treatment. Strategies to increase uptake of PrEP among BMSM should utilize a bottom-up approach, such as a community-engagement approach, and allow community members to play prominent roles in developing, vetting, and implementing PrEP campaigns and ensure messages can better address the needs and priorities of the community.

Crowdsourcing is “an approach to problem solving which involves an organization having a large group attempt to solve a problem or part of a problem, then sharing solutions [2].” Open contests [11], a form of crowdsourcing, allow members of a community to provide solutions to the problem in the form of a contest, and the crowdsourcing organization processes and consolidates these solutions into a unified product [12]. The solutions are expected to be highly relevant to the community because open contests explicitly incorporate local knowledge, culture, and style by directly involving a large number of community members in developing, vetting, and implementing ideas [13]. Open contests have collected new ideas or directions that address public health problems, such as identifying food deserts [14], improving sanitation [15], and sharing public health policy [13]. Multiple randomized controlled trials have provided evidence of the effectiveness of utilizing crowdsourcing approaches to improve sexual health [16-18]. This approach has yet to be fully realized as a method for improving the health and well-being of historically marginalized populations such as MSM. A recent scoping review stated that additional studies evaluating crowdsourcing to improve PrEP uptake are needed [19].

To tap into the community’s attitudes and values, we will implement a crowdsourcing open contest to solicit PrEP promotion messages for MSM in Baltimore and will evaluate the persuasiveness of the crowdsourced messages. This paper describes the research protocol and explores the feasibility and acceptability of a crowdsourcing open-contest approach to soliciting PrEP promotion messages.

## Methods

### Overview

This open contest will be implemented in Baltimore City, Maryland. National HIV Behavioral Surveillance data in 2017 indicated that the HIV prevalence among BMSM was 44% in Baltimore [20]. Less than two-thirds (63%) of HIV-negative BMSM were aware of PrEP as compared with 87% of white MSM. Only 14% of BMSM reported taking PrEP in the past year as compared with 21% of white MSM.

Open-contest implementation and evaluation will proceed as follows: (1) organize a community steering group; (2) develop platforms to solicit crowd input; (3) engage the community to contribute ideas through a Web-based forum and in-person events; (4) evaluate contest entries using both community panel judge assessment and crowd voting; (5) use mixed methods to evaluate feasibility, acceptability, and community engagement; and (6) disseminate contest results.

### Organize a Community Steering Group

A community steering group will be formed to represent various community-based organizations (CBOs), advocacy groups, and individuals who represent diverse segments of MSM communities. Members of the community steering group will be involved in every stage of the proposed study. The community steering group will meet bimonthly to provide guidance on how to promote the open contest to the communities and to assist with crowd recruitment strategies. The community steering group will also give suggestions for the study website design, contest prize structure, contest entry evaluation, and plans to disseminate findings.

### Develop Platforms to Solicit Crowd Input Through Both a Web-Based Forum and In-Person Events

We will work with our crowdsourcing technical partner, Community Expert Solutions, to organize a customized website where the crowd can submit their ideas. Before launching the Web-based forum, an evaluation of the acceptability of the specific features of the website and effective strategies to recruit participants online will be conducted and assessed among members of the community steering group.

On the basis of our previous experience [21] and feedback from community members, complementing Web-based activities with strong in-person activities is an essential component of organizing more effective and inclusive contests [22]. In-person activities are intended to reach marginalized groups with limited internet access and are key to building a rapport and trust with local partners and contributors. Collaborating with local CBOs, we will participate in various community events to promote and explain the rules of the contest. Individuals can submit their ideas during in-person events. Information about the open contest, including the call for challenges, criteria for judging, and contest prize structure, will be posted on the project website and distributed during in-person events.

Complex tasks, such as designing PrEP promotion campaigns, need to be broken down into small and discrete components to increase the probability that individuals will be able to contribute to the campaign. In this study, we will focus on generating novel content that can increase awareness and uptake of PrEP among BMSM in Baltimore. Content of interest in the current contest will be (1) hashtag (#) of the PrEP campaign and (2) a 1- or 2-sentence *call for action*. The criteria for judging will be shared with the crowd, but no examples will be provided to avoid cognitive fixation, which has been found to be a pervasive impediment to developing innovative ideas [23-25]. A contest prize structure will be developed based on the standard principles of the inducement prize contest theory [26] and with input from the community steering group. We will include both monetary and nonmonetary prizes and avoid focusing exclusively on the *winners*. The top 3 finalists will work with a team of experts to develop a final product of PrEP promotion posters.

### Engage the Community to Contribute Ideas

We will design the open contest to be as inclusive as possible. The eligibility to participate in the contest will be limited to those who are aged 18 years or older because of human subject restrictions on involving minors in research. Individuals will be able to submit more than one entry. All individuals who submit contest entries will provide a Web-based informed consent and fill out a brief survey that includes questions on sociodemographics, sexual identity, and PrEP awareness and use. To elicit contest entries that are most likely to be relevant to local BMSM, we will implement a target recruitment of BMSM. Potential participants for the open contest will be recruited from a venue-based outreach (within CBOs and at community events), social media (project’s Facebook, Instagram, and Twitter accounts), and word-of-mouth referral*.* Our recruitment team has developed a presence and strong partnerships with local communities and service agencies. There are no preestablished set points for knowing when a sufficient crowd threshold has been reached to yield crowd wisdom [27]. We will keep the contest submission open for 3 months and closely monitor the number of submissions. On the basis of our previous experiences with similar contests [19,21], we anticipate receiving 100 entries in 12 weeks.

### Evaluate Contest Entries and Announce Finalists

We will implement a 2-step approach to select the top finalists. A community panel of judges will first evaluate deidentified submissions to select the top-10 finalist submissions. The judging panel will include community steering committee members, health communication experts, public health officials, and CBO staff. We will create a mechanism for the judges to remove themselves based on conflict of interest (ie, any financial, organizational, or other interest that could be perceived). We will then host an open call for the public to vote on the top-10 finalist submissions. We will create a poll hosted on the contest website to enable users to vote for their favorite submission. The top-3 finalists will be selected based on the evaluation from the panel of judges and the popular votes.

After consulting with the community steering committee, we will host a public announcement of the 3 finalists, potentially in concurrence with other community-driven events, such as gay pride events in Baltimore. No identifiable information will be released without individual consent. Wider participation from individuals and CBOs will also be recognized to increase awareness of pressing issues among key populations with hard-to-reach groups. This acknowledgment of contribution is critical because most individual submissions will not be awarded prizes. We will ask participants about continued engagement for future open contests so that the end of the first contest also serves as the beginning of a process of sustained community engagement [26].

### Evaluate Feasibility, Acceptability, and Community Engagement

To explore the feasibility, acceptability, and community engagement of the open contest, we will conduct semistructured interviews with a purposive sample of 25 participants who (1) have participated in the open contest, (2) voted in the open contest, or (3) helped to organize the contest (eg, the in-person event). A preliminary interview guide will include major topics regarding expectations of participating in the contest, facilitators and barriers to contest participation, engagement in contest activities, and participants’ experience throughout the entire process of the contest. The preliminary interview guide will be pilot tested with 3 to 4 participants from the community. Feedback will be subsequently incorporated into the interview guide after discussion with the steering group and principal investigator. The interview questions will be open ended to allow participants to share their own ideas and expand on issues that are important to them. The interviews will be conducted by 2 trained qualitative researchers. All interviews will be audio-recorded, transcribed verbatim, reviewed for accuracy, and uploaded to Atlas.ti 7.1 (ATLAS.ti Scientific Software Development GmbH) for coding. The coding scheme will be developed using an iterative and collaborative process. Furthermore, 2 research team members will first review each transcript, and an initial coding framework will be formed based on predetermined questions that are used to design the in-depth interview guide and identified emergent themes. Coded quotes will be then chosen by group consensus to illustrate the variation and most common responses within each theme. After discussion among investigators, the coding scheme will be revised to ensure the coding process incorporates a range of perspectives [28]. The final coding scheme will then be applied to the remaining interviews. One of the goals of this analysis is to expand the literature by examining the process of organizing an open contest, differentiating stages of contest engagement, and examining how contest engagement may influence behaviors.

We will also evaluate the open contest by collecting online engagement statistics. Online engagement measures will be extracted using Facebook, Instagram, and Twitter analytics for the project’s social media sites and Google Analytics for the contest website. The measures of active online engagement include contest submissions, page follows (unique users who subscribed to page update alerts), page visits (unique users who visited a page), and demographic (age range and sex) and geographic (city and country) information about unique users who visit the social media pages or contest website. Finally, the reach will be measured by the number of unique users who passively viewed any posted content from the contest’s Twitter, Facebook, or Instagram pages. We will also examine the number of votes for each submission, page visits, and geographic information. These metrics have been used to examine the scope of community engagement for previous crowdsourcing contests [21].

### Dissemination

At the end of the study, the findings related to the open contest will be disseminated to the community steering committee, CBOs, and local health departments. These materials will include information needed to implement a crowdsourcing open contest, common barriers and issues experienced by participants, a protocol for needs assessments, and training materials. We will also present the study findings to HIV-prevention scientists through publications and conference presentations. We will also deposit the messages and materials in an open access database.

## Results

This study is funded by National Institutes of Health (National Institute of Mental Health: R34MH116725) in May 2018 and was approved by institutional review board in April 2018. The open contest started in February 2019, and data analyses for the mixed method evaluation are expected to complete in December 2019.

## Discussion

The study will make important contributions to the literature on crowdsourcing and health promotion among BMSM. This project will result in the creation of BMSM community-engaged PrEP promotion campaign messages that will be fielded and evaluated in an urban setting. We will determine whether a crowdsourcing open-contest approach is acceptable among BMSM in Baltimore. PrEP promotion messages selected as the top finalists can serve as a template for future contests that will focus on visual images (eg, color photographs, black-and-white photographs, and videos of less than 1 min) and distribution networks (eg, social media networks, in-person businesses, and in-person social networks relevant to BMSM). The sequential organization allows us to utilize different types of crowd talent while linearly building on the overall theme. The open-contest processes are applicable to organizations or programs regardless of the size of the organization or program or resources available, thus allowing scalability. Evaluation data from this study will inform similar future studies among BMSM that will be conducted in the United States. The findings of this proposed study will potentially bring new ideas to Baltimore City for the development of more impactful PrEP campaigns. By partnering closely with local CBOs, this project will give members of the BMSM community the tools and capacity to perform similar activities and further strengthen the relationship between CBOs and the local government. Finally, this approach could be used to identify and implement programs and policies that are more responsive to community needs and build up existing community assets, which is critical for strengthening health systems [29].

## Appendix

Multimedia Appendix 1 Resume and summary of discussion of NIH peer review.

## Abbreviations

BMSM: black men who have sex with men
CBO: community-based organization
MSM: men who have sex with men
PrEP: pre-exposure prophylaxis
