# Supplementary material for: A Crowdsourcing Open Contest to Design Pre-Exposure Prophylaxis Promotion Messages: Protocol for an Exploratory Mixed Methods Study
Source: JMIR Res Protoc. 2020 Jan 3;9(1):e15590. doi: 10.2196/15590 (PMC6969383; doi:10.2196/15590)
Supplement: Multimedia Appendix 1 [file resprot_v9i1e15590_app1.pdf]

1R34MH116725-01 Yang, Cui

RESUME AND SUMMARY OF DISCUSSION: The applicant addresses the issue of low PrEP uptake among men who have sex with men (MSM) in Baltimore by proposing to investigate the feasibility, acceptability, and utility of an open contest approach to develop PrEP relevant messages and a neurocognitive approach – functional near-infrared spectroscopy (fNRIS) – to determine the persuasiveness of the messages. This is a rather innovative study both in the use of open contest to select PrEP messaging and fNRIS as a tool to assess their influence. The applicants are outstanding, having the complementary skills to accomplish near every aspect of the research. The premise of this application is strong as studies have demonstrated that neural responses may be significantly more effective in predicting behavior than self-report. The focus on MSM in Baltimore is important; awareness of PrEP among MSM as an effective HIV prevention intervention is very low; despite marketing efforts expanded by the health department, only 3% of this population was aware of a recent PrEP campaign launched by the Baltimore City Health Department (BCHD). An assessment of their perception of that campaign revealed that a large number did not feel that it resonated with them. The application has many strengths: it is well written, innovative; the hypotheses and analysis plans are clear, and the team has most of the expertise necessary to conduct this project successfully. Some weaknesses were raised; they included the following: there is a lack of expertise in neuropsychology on the team; there was no discussion of potential effects of age, medication use, education, history of drug dependence on fNIRS; they are only targeting the prefrontal cortex when other brain regions may be involved due to variety of task demands, and some RCT objectives might be a little vague. Nevertheless, these were considered minor, especially in the context of an R34, and the committee was unanimously excited about an application which they considered cutting-edge and potentially capable of informing the design of effective marketing campaigns.

DESCRIPTION (provided by applicant): With the release of Pre-Exposure Prophylaxis (PrEP) clinical guidelines by the CDC, there has been increased effort to identify effective strategies for increasing awareness, acceptability, and uptake of PrEP as a HIV primary prevention tool, especially among men who have sex with men (MSM). Conventional approach to developing HIV campaigns, such as social marketing, is fundamentally a “top-down” approach by designing programs created by select individuals from a key population. In addition, most pre-testing of campaign messages is done using self-report methods, such as focus groups or likeability measures. Although these methods are useful, research has found that self-report effectiveness measures cannot reliably predict the effectiveness of campaign materials. Evidence from neuroscience has demonstrated that neural responses hold the potential to be significantly more reliable than self-report measures in assessing message effectiveness that is highly predictive of future behavior change. This proposal will examine the feasibility, acceptability, and utility of open contest approach and neuroimaging technique to develop and evaluate PrEP promotion messages for high-risk MSM in Baltimore, MD. Our multi-disciplinary research team brings together HIV prevention, open contest, and neuroimaging experiment experience; a long term history of working with MSM in Baltimore; and a rich local infrastructure for HIV research. The specific aims are: 1) Explore the feasibility and acceptability of open contest approach to soliciting PrEP promotion messages, and 2) Evaluate the effectiveness of PrEP promotion messages developed via open contest by assessing neural bases of persuasion. Findings of this proposed study will transform the design, evaluation, and implementation of HIV campaigns, potentially bringing new ideas for local health departments and community-based organizations in developing more impactful PrEP campaigns.

PUBLIC HEALTH RELEVANCE: The proposed study is relevant to the persistent high HIV incidence and prevalence rates among MSM. This proposal will examine the feasibility, acceptability, and utility of open contest approach and neuroimaging technique to develop and evaluate PrEP promotion messages for high-risk MSM. Findings of this proposed study will transform the design, evaluation, and implementation of effective PrEP promotion campaigns.
